# Explosive Contagion in Networks

**DOI:** 10.1038/srep19767

**Published:** 2016-01-28

**Authors:** J. Gómez-Gardeñes, L. Lotero, S. N. Taraskin, F. J. Pérez-Reche

**Affiliations:** 1 Institute for Biocomputation and Physics of Complex Systems (BIFI), University of Zaragoza, Zaragoza, Spain; 2 Department of Condensed Matter Physics, University of Zaragoza, Zaragoza, Spain; 3 Departamento de Ciencias de la Computaci´on y de la Decisión, Universidad Nacional de Colombia, Medellín, Colombia; 4 St. Catharine’s College and Department of Chemistry, University of Cambridge, Cambridge, UK; 5 Institute for Complex Systems and Mathematical Biology, SUPA, King’s College, University of Aberdeen, Aberdeen, UK; 6 Facultad de Ingeniería Industrial, Universidad Pontificia Bolivariana, Medellín, Colombia

## Abstract

The spread of social phenomena such as behaviors, ideas or products is an ubiquitous but remarkably complex phenomenon. A successful avenue to study the spread of social phenomena relies on epidemic models by establishing analogies between the transmission of social phenomena and infectious diseases. Such models typically assume simple social interactions restricted to pairs of individuals; effects of the context are often neglected. Here we show that local synergistic effects associated with acquaintances of pairs of individuals can have striking consequences on the spread of social phenomena at large scales. The most interesting predictions are found for a scenario in which the contagion ability of a spreader decreases with the number of ignorant individuals surrounding the target ignorant. This mechanism mimics ubiquitous situations in which the willingness of individuals to adopt a new product depends not only on the intrinsic value of the product but also on whether his acquaintances will adopt this product or not. In these situations, we show that the typically smooth (second order) transitions towards large social contagion become explosive (first order). The proposed synergistic mechanisms therefore explain why ideas, rumours or products can suddenly and sometimes unexpectedly catch on.

Communication between pairs of individuals constitutes the basic building block of macroscopic contagion and dissemination of social phenomena such as behaviors, ideas or products. The mathematical formulation for social diffusion is reminiscent of the spread of infectious diseases and it is indeed common to use the term *viral* to refer to the rapid advent of a product or an idea. Following this analogy, compartmental epidemic models such as the Suceptible-Infected-Susceptible (SIS) or the Susceptible-Infected-Recovered (SIR) are often used to describe the dynamics of the transmission of social phenomena^1^,^2^,^3^.

Epidemic models assume that the transition to macroscopic epidemic invasions in a population can be fully explained in terms of microscopic contagions between pairs of individuals. However, the dynamics of social transmission do not only depend on the characteristics of the transmitting and receiving individuals (*e.g.* on attitude or persuasiveness) but also depend on the context of the transmission event. In particular, individuals connected in some way to transmitter-receiver pairs of individuals might have important and unexpected effects on the spread of social phenomena at the global population level^4^,^5^.

The first attempt to include the influence of the context within an epidemiological modelling framework was made by Daley and Kendal (DK)^6^. In the DK model, an individual spreading a rumor or idea may stop spreading it and become a stifler after realizing that the rumor is already known by some of its contacts. The importance of accounting for this effect was highlighted in their work by showing that a rumor can reach a large fraction of a population even if it is transmitted at an infinitesimally small rate *α*. This finding was in sharp contrast with prototype SIR epidemics which ignore the effects of individuals surrounding infected-susceptible pairs and only predict large invasions if the rate of transmission of infection is larger than a certain critical value, *i.e.* if 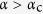
7. Despite the different location of the invasion threshold given by the DK and SIR models, both models and their variants^8^ predict that the number of individuals affected by the spreading phenomenon increases smoothly with increase of the pair transmission rate, *α*. This corresponds to a *second-order phase transition* from non-invasive to invasive regime at the critical value, 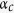. Continuous transitions were also obtained with an extended SIR model involving context-dependent transmission mechanisms assuming that each pairwise contagion can be enhanced or diminished depending on the number of infected/spreader individuals surrounding the transmitter-receiver pair^9^,^10^.

A continuous transition between the non-invasive and invasive regimes is not able to explain the fact that social phenomena often become accepted by many people overnight. Examples include the sudden unfolding of social movements or the rapid increase in popularity of new communication tools^11^. Such explosive contagions would correspond to a first-order phase transition from non-invasive to invasive regimes in which the number of individuals affected by the spreading phenomenon exhibits a discontinuous increase. Explosive transitions to large contagion have been predicted by some models incorporating complex synergistic mechanisms. These include transmission dynamics in which ignorants can only become spreaders if they are surrounded by a number of spreaders larger than a certain threshold^12^,^13^,^14^ and models in which transmission is enhanced by constructive memory of ignorants to previous exposures to the spreading phenomenon^15^,^16^,^17^,^18^,^19^ or by a non-linear cooperation of the transmitting spreaders^20^,^21^. Note that weakly non-linear and memory-less synergistic transmission mechanisms studied in refs 9,10 do not lead to explosive transitions. This suggests that strong non-linearity and memory to previous transmission attempts are important factors leading to explosive transitions. Explosive transitions have also been observed in models which assume adaptive rewiring of contacts of susceptible hosts to avoid infection from infected individuals^22^. In this case, rewiring plays a crucial role for explosive transitions since eliminating contacts without further rewiring leads to continuous transitions^23^.

Models predicting explosive contagion typically assume strong synergistic effects involving receivers (ignorant individuals) and transmitters (spreaders); the effects of ignorant acquaintances of receivers are typically neglected. In this article, we show that explosive transitions can also occur when the acquaintances of ignorant receiver individuals are highly reluctant to accept new social phenomena. This seemingly paradoxical result is especially relevant to social contexts in which individuals hesitate joining a collective movement, *e.g*. a strike, fearing the risk of becoming part of a minority that can eventually be punished. This scenario also corresponds to typical social settings. For instance, social media such as YouTube, Facebook or Whatsapp typically have a very fast acceptance^11^ which depends on both its intrinsic value and perceived value given by our acquaintances.

## Synergistic transmission rate

**Figure 1:**
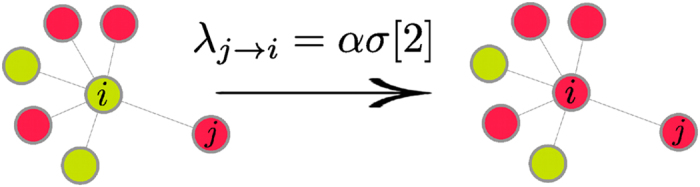
Schematic plot of the transmission from a transmitter *j* to a receiver *i* with synergistic rate given by Eq. (1) when there are 2 ignorant/healthy individuals (green circles) surrounding *i*.

The model introduced here extends those proposed in refs 9,10 to incorporate the effects of ignorant individuals connected to receivers (see Fig. 1). Note that this contrasts with the mechanisms used in refs 9,10 which focused on synergistic effects of spreaders attached to receivers. In particular, we model the transmission rate, 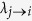, from a transmitter *j* to an ignorant/healthy receiver *i* as:


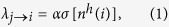


where *α* accounts for the intrinsic value of the spreading phenomenon in the absence of the context. The number, 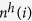, of ignorant/healthy individuals connected with the receiver, *i*, can affect transmission from *j* to *i* and this is accounted for by the function 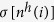. Non-synergistic models with constant transmission rate, 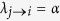, are recovered for 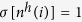. We analyse the effects of synergistic transmission using two representative cases for the function 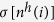: (i) exponential,


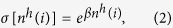


and (ii) linear dependence on 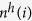,


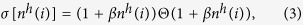


where 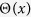 is the Heaviside theta-function which takes the values 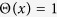 for 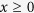 and 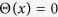 for 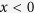. The parameter *β* quantifies the constructive 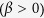 or interferring 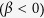 synergy effect of 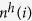 on transmission. The exponential dependence assumed in Eq. (2) offers a convenient way to ensure that 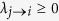 for any value of *β*. We therefore use this form to illustrate most of our results. However, use of linear synergistic rates leads to qualitatively similar results and conclusions (See the Supplementary Information).

## Explosive contagion in SIS epidemics

The evolution of the spreading process depends both on transmission rates and dynamical rules imposed. For concreteness, we start the analysis by employing the exponential synergistic transmission rates (2) for contagion dynamics given by the rules of the SIS epidemic model applied to a population of *N* individuals. The individuals form a network of contacts through which information spreads. To start with, we illustrate our results by using an Erdös-Rényi (ER) graph of size 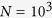 with a Poisson degree distribution, 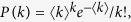 characterised by mean node degree 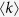. Below we report similar phenomenology for *k*-regular graphs.

In the SIS dynamics, each individual can be either *susceptible* (ignorant) or *infected* (spreader). Within discrete-time transmission dynamics employed in most of our simulations, a spreader, *j*, in a time step 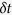 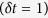, can either transmit the social phenomenon to an ignorant, *i*, with probability 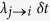 or can become ignorant with probability 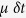. Starting from a population composed of ignorants and a small number of spreaders, 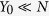, the number of spreaders, *Y*, evolves in time and the system reaches a quasi-steady state which can either be free of spreaders (spreader-free state characterised by *Y* = 0) or correspond to an endemic state with a positive number of spreaders, 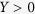, coexisting with 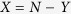 ignorants. Coexistence of *Y* and *X* in the endemic steady-state is a consequence of a balance between the new infections occurring at each time step and the number of individuals becoming ignorant. The endemic invasive regime appears when 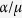 takes large enough values.

**Figure 2:**
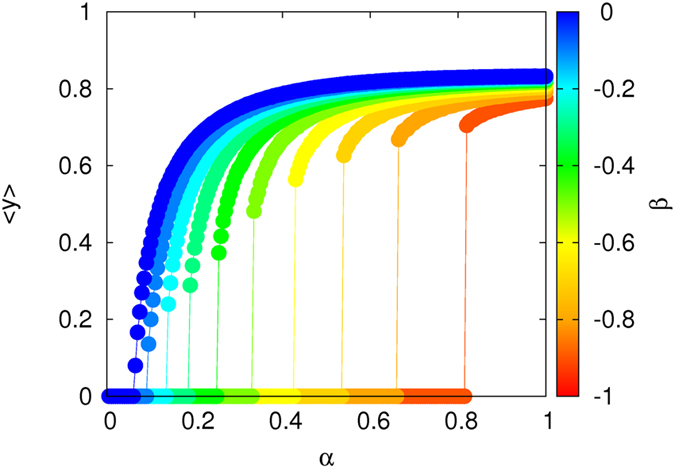
Concentration of spreaders,〈*y*〉, in the steady state of SIS epidemics on Erdös-Rényi networks with〈*k*〉=4 as a function of the inherent transmission rate, *α*. Different curves correspond to different values of the synergistic parameter, *β*. The recovery rate of spreaders is 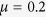.

Figure 2 shows the concentration of spreaders in the steady state, 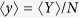, as a function of *α* for several values of the synergistic parameter *β*. The curves shown are calculated as follows. For each value of *β*, the simulation starts with 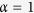 from a configuration in which a small fraction (around 5%) of the nodes is initially set randomly as spreaders and the rest are ignorant. For each value of *α*, we iterate the dynamics for a large number of time steps so that 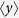 can be accurately measured. Subsequently, *α* is decreased by 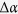 and the Monte Carlo (MC) simulation starts again, taking as initial conditions the last configuration obtained for the previous value of *α*. In this way, we perform an *adiabatic continuation* to compute each of the 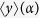 curves shown in Fig. 2.

The striking result is that, for negative enough values of *β*, the synergistic SIS model displays an abrupt phase transition from the spreader-free (healthy) phase to the endemic one. This explosive onset of the endemic regime is our main finding and it is in sharp contrast with the results obtained with the traditional non-synergistic epidemic models.

## Markovian microscopic evolution

Additional evidence for the phenomenon can be obtained by numerical solution of the Markovian microscopic evolution equations extending the method introduced in^24^,^25^ by incorporating the synergy effects. The key quantities in this approach are the probabilities 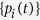 that an individual *i* is a spreader at time *t*. Their evolution is given by the following equations:


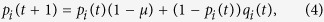


where 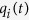 is the probability that an ignorant node, *i*, gets in contact with a neighbouring spreader neighbour and becomes a spreader itself:


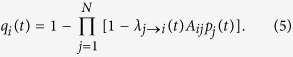


Here, 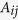 is the 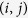-th component of the adjacency matrix defined as 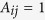 if nodes *i* and 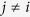 are connected and 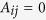 otherwise. The probability of infection 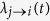 is a time- and context-dependent variable which we approximate by


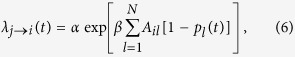


using the expression 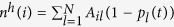 for the number of healthy neighbors of a node *i* at time *t*. By solving the set of Eqs. (4), one obtains the stationary distribution 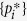 that yields the stationary value of infected individuals 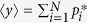.

**Figure 3:**
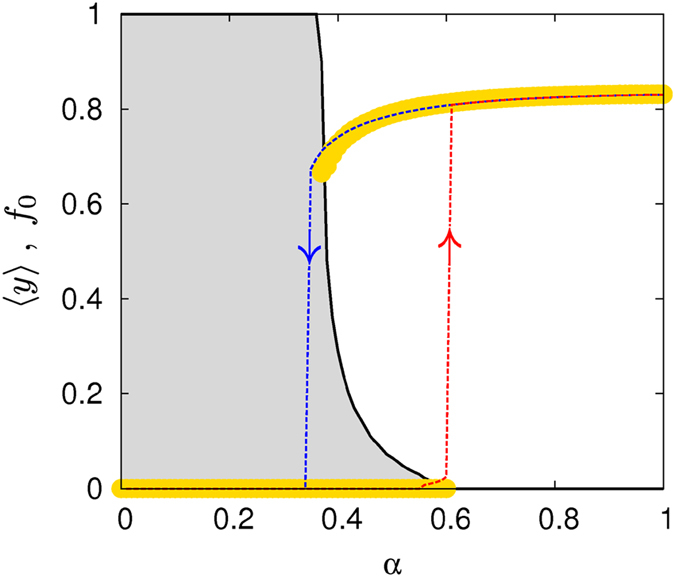
Concentration of spreaders,〈*y*〉, as a function of *α* for the SIS process in an Erdös-Rényi network of〈*k*〉=6 when *β* = −0.5. The dashed curves indicate the solution obtained by solving the Markovian evolution equations whereas the solid amber circles correspond to the results obtained by using MC simulations (10^3^ realizations for each value of *α*). The hysteresis effect points out the existence of a bi-stability region. The solid curve shows the fraction 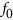 of ralizations (in the MC simulations) that end up in the fully ignorant solution, 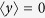. The recovery rate is 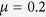.

In Fig. 3, we show the results of the numerical solution of Eqs. (4) for 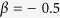 in an ER network of mean degree 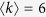. Eqs. (4) have been solved by considering two different sets of initial conditions corresponding to either 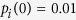, 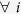 (the red dashed curve with an up-arrow) or 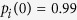, 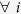 (the blue dashed curve with a down-arrow). For small and large values of the inherent contagion rate, *α*, the solutions are independent of the initial conditions. In contrast, two different stationary states corresponding to the spreader-free 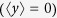 and endemic 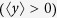 regimes are observed for 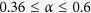 depending on the initial conditions. Thus, both the MC and Markovian evolution predict the coexistence of endemic spreading and spreader-free states and the corresponding hysteresis effect with discontinuous transitions between these regimes.

The above results are corroborated by MC simulations run from different initial configurations with fractions of spreaders drawn uniformly at random between 0 and 1 (in contrast to data presented in Fig. 2 where, due to particular choice of initial conditions, only the upper branch of the hysteresis in the bi-stability region is displayed). The comparison between the two approaches is also shown in Fig. 3 in terms of the fraction 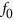 of initial configurations leading to the spreaders-free regime in MC simulations (see the continuous line in Fig. 3). The bi-stable region predicted by the Markovian formalism is indeed well captured by the region where 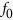 changes between 0 and 1.

## Mean-Field model

To gain further insight on how explosive transitions appear in the synergistic SIS model, we consider a heterogeneous mean-field model. Within this formalism, the concentration, 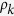, of spreaders of degree *k* evolves as follows^26^:


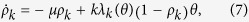


where 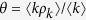 is the average fraction of spreaders surrounding each node. The rate of transmission towards an ignorant *i* of degree *k* is given by 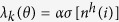 which is a function of the average number of ignorant nodes, 
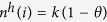
, surrounding the receiver, *i*.

The stationary state of the SIS process in mean-field approximation corresponds to the condition 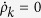, 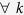 which, from Eq. (7), satisfies the following condition:


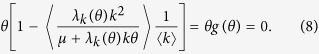


This equality is trivially satisfied for 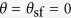 which corresponds to the spreader-free regime. The non-trivial regime with macroscopic spreading corresponds to 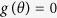. Eq. (8) can be solved analytically for a network with a random *z*-regular graph topology characterised by a degree distribution, 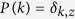. In this case, the concentration of spreaders, *y*, coincides with *θ* which is the solution of 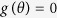. The later condition can be recast for *y* in the following form:


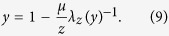


The solution of Eq. (9) for exponential synergistic transmission rate, 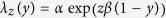 (a linear rate leads to analogous results as shown in the Supplementary Information), is 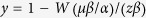 when 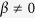 and 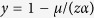, otherwise. Here, the Lambert function, 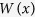, is implicitly defined by the relation 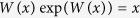
27.

The Lambert function is only defined for 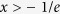 and, importantly, it is double-valued in the interval 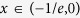. The condition 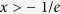 implies that systems with inherent transmission rate 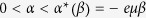 are necessarily in the spreader-free regime with 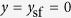. For 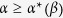, there are two non-trivial solutions associated with the two branches, 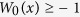 and 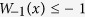, of 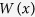 for 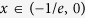, with the physical solutions being in the range 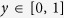. The analysis of the two branches of 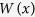 reveals that the transition from non-invasive to invasive regime when increasing *α* at fixed *β* is smooth if 
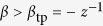
 since only the branch 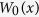 leads to positive values of *y*. This occurs for:


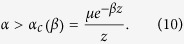


Here, 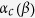 is an epidemic threshold corresponding to the situation in which the positive non-trivial solution to Eq. (9) coincides with the spreader-free solution, i.e. 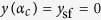. Note that for 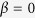 the usual threshold for the SIS process is recovered at 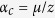. For 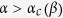, the solution 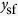 becomes unstable while the positive solution for *y* is stable and corresponds to the endemic state.

Explosive transitions are observed for 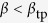 where the two branches of 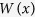 take positive values as soon as 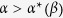. However, the solution corresponding to 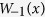 becomes negative for 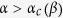 and must be discarded. We then conclude that the bi-stability region associated with the explosive transition is restricted to values of 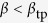 and 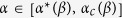. Finally, the mean field analysis concludes that the three possible regimes (epidemic, healthy and bi-stability) meet at a tricritical point^28^ located at:


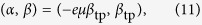


where the invasion transition occurring with increasing *α* at fixed *β* changes from second- to first-order with decreasing *β*.

**Figure 4:**
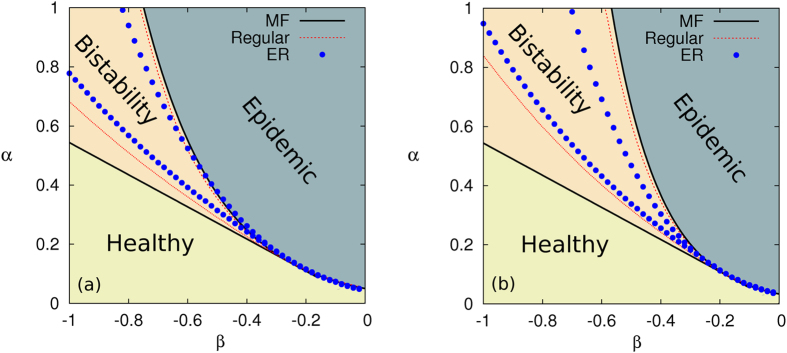
Contagion diagram in the (*α*, *β*) plane. The solid curves show the theoretical mean-field prediction for the boundaries of the bi-stability region, 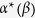 and 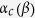, in a random *z*-regular graph with (a) 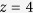 and (b) 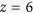. The dashed lines and circles show the corresponding boundaries computed by solving the Markovian evolution equations in a *z*-regular graph and an ER network with 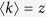, respectively. The recovery rate is set to 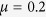 in both panels.

In Fig. 4, we show the contagion diagram in the 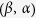 plane. The solid curves show the analytical predictions 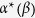 and 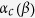 for random *z*-regular graphs with 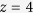 and 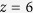 in panels (a) and (b), respectively. The results are in good agreement with the bi-stable region obtained by solving the Markovian evolution equations for *z*-regular graphs (see dashed curves in Fig. 4). In addition, the circles display the boundaries for ER graphs with 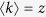. It becomes clear that the node degree heterogeneity of ER graphs leads to a smaller bi-stability region compared with the prediction for random *z*-regular graphs. On the other hand, the position of the triple point in ER networks (*i.e.* the intersection of the two branches of circles) is in good agreement with the theoretical and numerical values (located at the intersection of solid and dashed curves, respectively) obtained for *z*-regular graphs.

## Explosive contagion with removal of spreaders

The SIS model assumes that spreaders may temporarily stop spreading the social phenomenon but can eventually resume spreading it after meeting a spreader. In some cases, however, it can be more appropriate to assume that spreaders cease spreading permanently, *i.e.*, they become stiflers or removed by passing from the spreader state to a new compartment for removed individuals, as in the SIR epidemic model. Within a mean-field framework, it is possible to formulate a model with rather general removal mechanisms which encompass both the SIR model and a variant of the DK model introduced by Maki and Thompson (MT)^29^. The dynamics of the concentrations of ignorants (*x*), spreaders (*y*) and removeds (*r*) on random *z*-regular graphs are given by the following equations:


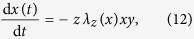



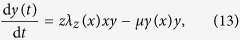



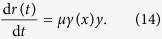


These equations assume that the population remains constant, *i.e.*, the concentrations satisfy the closure condition 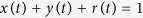 for every *t*.

The transmission rate is defined as 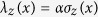, where 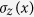 gives a synergistic contribution to transmission which depends on the number of ignorants, 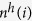, surrounding a receiver *i*, i.e. 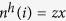 (Table 1 gives expressions of 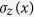 for the cases of exponential and linear synergistic transmissions).

Finally, the transition from the spreader state to the removed one is mediated in Eqs. (13) and (14) by parameter *μ* (the spontaneous removal rate of a spreader) and the function 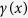 that captures several possible mechanisms for removal of spreaders. In particular, the SIR model assumes that spreaders stop spreading the social phenomena spontaneously (*i.e.* removal is not affected by encounters with other individuals). In contrast, the MT model assumes that recovery can only occur when a spreader meets another spreader or a removed individual (*e.g.* a stifler). These two behaviours can be modelled by setting (cf. Table 1),


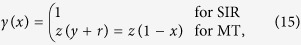


so that the analysis of SIR and MT models can be done in a unified way by solving Eqs. (12)–(14), 118, 119.

In general, it is not possible to obtain an exact solution to the system defined by Eqs. (12)–(14), 118, 119. However, it is possible to obtain the final concentration of removed individuals, 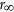, which quantifies the reliability of any spreading phenomenon with permanent removal of spreaders. The solution is given in implicit form by the following equation which is more conveniently expressed in terms of the final concentration of ignorants, 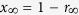 (see the Supplementary Information for details):


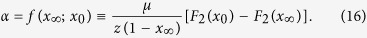


Here, 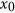 is the initial concentration of ignorants and the function,


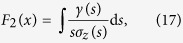


incorporates synergistic and removal mechanisms governed by 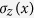 and 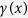, respectively. Particular expressions for 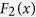 corresponding to different removal and synergistic mechanisms analysed in this work are given in Table 1. Explosive contagion transitions occur when Eq. (16) gives more than one solution for 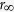. The regimes with continuous and explosive transitions are separated by a critical regime for which 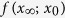 displays an inflection point at some value of 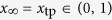. These conditions and definition of 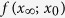 given by Eq. (16) result in the following equations for the tricritical point:


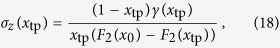



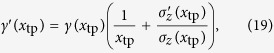


where the prime denotes the derivative with respect to *x*. From Eqs. (18) and (16), the inherent transmission rate at the triple point can be expressed as:


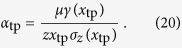


In general, any spreading phenomenon with removal of spreaders for which Eqs. (18)–(19), 141 have a solution with 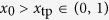 can exhibit explosive transitions for strong enough interfering synergy. In particular, both the SIR and MK model exhibit explosive transitions, in analogy to those exhibited by 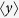 in the SIS model. In the Supplementary Information, we present a complete analysis of the general equations derived here for the SIR model with linear synergistic transmission rate. Despite being a relatively simple model, it exhibits the main typical features of explosive transitions characteristic of more complicated models.

**Figure 5:**
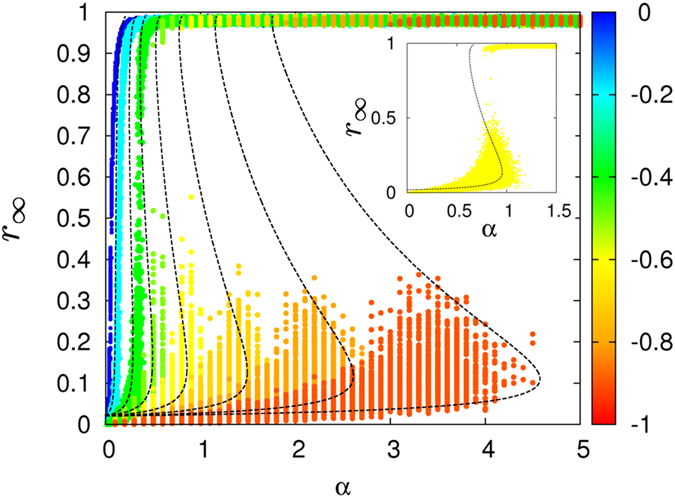
Concentration of removeds at the end of SIR epidemics as a function of the inherent transmission rate, *α*. The initial concentration of ignorants is 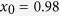 and the removal rate is 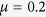. Symbols indicate the results of MC simulations for ER networks of size 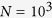 and 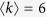 (10^3^ realizations of epidemics for each value of *α*; all the epidemics run on the same random graph). Different colours correspond to different values of *β*, as marked by the colour-box. Lines show the analytical solutions of the synergistic SIR mean-field model. A magnified view of the onset of the discontinuity for 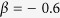 is displayed in the inset.

In Fig. 5 we show the solutions of Eq. (16) (dashed curves) for the SIR model with exponential synergistic rate together with the results (points) obtained by MC simulations. The evolution of the dashed curves reveals a transition from smooth to explosive regimes when decreasing *β*. These results correspond to a relatively large initial concentration of ignorants. However, it is possible to show that explosive transitions can be observed for any positive initial concentration of ignorants provided 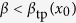, where 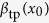 decreases with 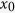. Remarkably, the discontinuous transitions predicted by the mean-field analysis are corroborated by the numerical MC simulations, showing bi-stability regions in which low and large reliability of the spreading phenomenon coexist in an interval of *α*.

## Discussion

In summary, our results give compelling evidence for explosive transitions towards macroscopic acceptance of social phenomena. The explosive nature of these transitions has important implications in real social scenarios. For instance, it may represent unexpected and challenging barriers for the control of global pandemics of undesired social phenomena or, conversely, an exciting scenario for the diffusion of innovative products and ideas. The key factor responsible for explosive transitions is the negative action on transmission of ignorant neighbours. Such opposition prevents transitions to large contagion until the transmission becomes strong enough as to overcome the reluctance of ignorant contacts. At this point, an explosion to large contagion occurs. Thus, explosive contagions appear as by-product of the inhibition of the epidemic onset up to a point in which a macroscopic avalanche of contagions unavoidably occurs. Note that inhibitory mechanisms are absent in our previous models where synergy was associated with infected neighbours of receivers^9^,^10^. We have checked that such synergistic mechanism leads to discontinuous transitions in SIS epidemics for sufficiently constructive synergy but transitions in SIR spread are continuous^9^,^10^. In contrast, synergy associated with ignorant neighbours leads to more ubiquitous explosive transitions which occur with and without removal of spreaders. Again, this highlights the important role of inhibitory mechanisms on explosive transitions.

The mechanism leading to explosive contagions is reminiscent of the cluster merging processes proposed in explosive percolation models^30^,^31^,^32^,^33^,^34^,^35^. However, these models rely on global external biases for cluster merging favouring the delay of the percolation transition which often lack a clear motivation and application^31^. In our case, explosive contagions result from the combined action of local synergistic effects, in line with the microscopic rules responsible for explosive synchronization phenomena^36^,^37^,^38^,^39^, jamming in complex networks^40^ or generalized epidemics^16^,^17^,^18^. We have shown that synergy associated with ignorant neighbours leads to genuine discontinuous transitions on random graphs involving a relative fraction of hosts smaller than one. This phenomenology is similar to discontinuous percolation transitions of type-II in cluster merging processes^35^.

Very recently, discontinuous transitions of this type have also been reported for contact processes^41^, in which the recovery mechanism is similar to that of the SIS model. Here we have shown that discontinuous transitions to global contagion are not only observed in SIS dynamics but are robustly predicted for models with permanent recovery of spreaders. Such models are arguably more realistic than SIS and contact processes for the spread of social phenomena. It is important to stress that, although non-linear effects in transmission rates can promote discontinuous transitions^20^,^21^, nonlinearity is not the driving force responsible for explosive contagions associated with inhibition by ignorant acquaintances, since they occur even for weakly non-linear synergistic rates.

Synergistic mechanisms studied here and in our previous works^9^,^10^ are associated with the number of ignorant neighbours of spreaders or the number of spreader neighbours of receivers, respectively. However, our models could easily be adapted to study the effects of other synergistic mechanisms associated with, e.g. the relative fraction of ignorant or spreader neighbours instead of their number^42^,^43^,^44^. Given the relatively low node degree heterogeneity of the networks considered in this work, we do not envisage qualitative differences between our results and those for a transmission rate depending on the fraction of neighbours. In contrast, differences might be more significant for spread in networks with more heterogeneous node degree (e.g. in scale-free networks^23^).

## Additional Information

**How to cite this article**: Gómez-Gardeñes, J. *et al.* Explosive Contagion in Networks. *Sci. Rep.* **6**, 19767; doi: 10.1038/srep19767 (2016).

## Supplementary Material

Supplementary Information

## Figures and Tables

**Table 1:** Summary of the functions describing the models with removal of spreaders.

Model	*γ*(*x*)	*σ*_*z*_(*x*)	*F*_2_(*x*)
SIR, no synergy	1	1	ln(*x*)
SIR, linear synergy	1	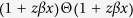	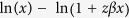
SIR, exponential synergy	1	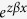	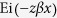
MT, no synergy	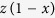	1	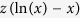
MT, linear synergy	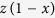	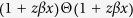	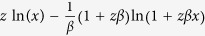
MT, exponential synergy	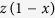	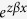	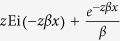

Expressions are given for random *z*-regular graphs. The function 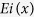 appearing in 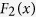 for models with exponential synergy is the exponential integral defined as 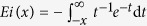.
