# Supplementary material for: Explosive Contagion in Networks
Source: Sci Rep. 2016 Jan 28;6:19767. doi: 10.1038/srep19767 (PMC4730159; doi:10.1038/srep19767)
Supplement: Supplementary Information [file srep19767-s1.pdf]

Explosive Contagion in Networks (Supplementary Information)

Jesús Gómez-Gardeñes,[1,2] Laura Lotero,[3] Sergei N. Taraskin,[4] and Francisco J. Pérez-Reche[5]

[1]*Institute for Biocomputation and Physics of Complex Systems (BIFI), University of Zaragoza, E-50018 Zaragoza, Spain*

[2]*Department of Condensed Matter Physics, University of Zaragoza, E-50009 Zaragoza, Spain*

[3]*Departamento de Ciencias de la Computación y de la Decisión, Universidad Nacional de Colombia, Medellín, Colombia.*

[4]*St. Catharine's College and Department of Chemistry, University of Cambridge, Cambridge, UK*

[5]*Institute for Complex Systems and Mathematical Biology, SUPA, King's College, University of Aberdeen, Aberdeen, UK*

SIS SYNERGY MODEL WITH LINEAR SYNERGY RATE.

In this section, we solve the SIS model exactly for a population of individuals having the network of contacts with the topology of random z-regular graph with linear dependence of transmission rate on the number of ignorant/healthy neighbours and demonstrate that this solution is analogous to that for the exponential dependence of $\sigma_z(n^h(i))$ discussed in the main text.

The equilibrium states correspond to the solutions of Eq. (6) in the main text which can be recast in the following form:

$$F_1(y) \equiv -y(\mu - z\,\lambda_z\,(1-y)) = 0 \ . \qquad (1)$$

In the synergy-free case, $\lambda_z = \alpha$ and the stable solution of Eq. (1) is $y = 0$ for $\alpha \le \alpha_{\rm c} = \mu/z$ and $y = 1 - \mu/(\alpha z)$ for $\alpha > \alpha_{\rm c}$.

In the model with linear synergy, the transmission rate is given by $\lambda_z = \alpha(1 + \beta z(1-y))$ and $F_1(y)$ is a third order polynomial in y which can have from one to three real roots. The equilibrium solution, $y_{\rm sf} = 0$ (spreader-free regime), is always present while the two other roots,

$$y_\pm = 1 + \frac{1}{2\beta z}\left(1 \pm \sqrt{1 + \frac{4\beta\mu}{\alpha}}\right) \ , \qquad (2)$$

are real only for $\alpha \ge \alpha^*(\beta)$, where

$$\alpha^*(\beta) = -4\beta\mu \ . \qquad (3)$$

The values of $y_\pm$ represent equilibrium concentrations of spreaders and thus must be in the range, $y_\pm \in [0,1]$. An equilibrium concentration, $y_{\rm eq}$, corresponding to a root of $F_1(y)$ can refer to either stable (if $F_1'(y_{\rm eq}) < 0$) or unstable (if $F_1'(y_{\rm eq}) > 0$) equilibrium.

In the $\alpha - \beta$ parameter space, there is a special (tricritical) point, $(\alpha_{\rm tp}, \beta_{\rm tp}) = (2\mu/z, -1/(2z))$ (see the point labelled by TP in Fig. 1), at which all three roots of $F_1(y)$ coincide, i.e. $y_{\rm sf} = y_- = y_+ = 0$. This point separates the regimes of explosive and continuous transitions between non-invasive (spreader-free) and invasive (endemic) epidemics. Fig. 2 shows the dependence of the equilibrium concentration of spreaders, $y_{\rm eq}$, on α for fixed value of β above (panel (a)) and below (panel (b)) the tricritical point. For fixed β above the tricritical point, $\beta > \beta_{\rm tp}$, and values of α smaller than critical value,

$$\alpha_c(\beta) = \frac{\mu}{z + \beta z^2} \ , \qquad (4)$$

both roots $y_\pm$ are outside the physical range $[0,1]$ and the only stable equilibrium at $y_{\rm sf} = 0$ corresponds to the spreader-free state (cf. Fig. 2(a)). For $\alpha = \alpha_{\rm c}(\beta)$ (see the solid line in Fig. 1), the root y_- intersects the allowed range $[0,1]$ at a point where $y_- = y_{\rm sf} = 0$. With increasing value of $\alpha > \alpha_c(\beta)$, the equilibrium concentration y_- continuously increases in the interval $[0,1]$ and it corresponds to the stable equilibrium ($F_1'(y_-) < 0$) while the spreader-free equilibrium, $y_{\rm sf} = 0$, is unstable ($F_1'(y_{\rm sf}) > 0$) for these values of α. This means that an increase in the inherent transmission rate at fixed $\beta > \beta_{\rm tp}$ drives the system continuously from spreader-free ($\alpha \le \alpha_c(\beta)$) to endemic ($\alpha > \alpha_c(\beta)$) state (the region above continuous line in Fig. 1).

For values of β below the tricritical point, $\beta < \beta_{\rm tp}$, the scenario is very different from that described above (see Fig. 2(b)). Indeed, if $\alpha < \alpha_*$, the only acceptable root of F_1 is $y_{\rm sf}$ which corresponds to the stable spreader-free state (the region below the dashed line in Fig. 1). At $\alpha = \alpha_*(\beta)$, the roots $y_\pm$ become real and take values in the range $(0,1)$, i.e. $0 < y_+ = y_- < 1$. With increasing α in the interval $\alpha \in (\alpha_*(\beta), \alpha_c(\beta))$ (the region between dashed and dot-dashed lines in Fig. 1) at fixed β, these two roots split in such a way that $0 < y_+ < y_- < 1$. The concentration y_- corresponds to the stable equilibrium while y_+ to the unstable one. Overall, there are two stable equilibria describing the spreader-free state with concentration of spreaders $y_{\rm sf} = 0$ and endemic state with concentration of spreaders equal to y_-. The finite gap between these two equilibrium states is a signature of discontinuous explosive transition between non-invasive and invasive epidemics. With further increase of α for fixed value of β, the root y_+ leaves the physical range $[0,1]$ when $\alpha = \alpha_c(\beta)$ (and $y_+ = 0$), and the only stable equi-

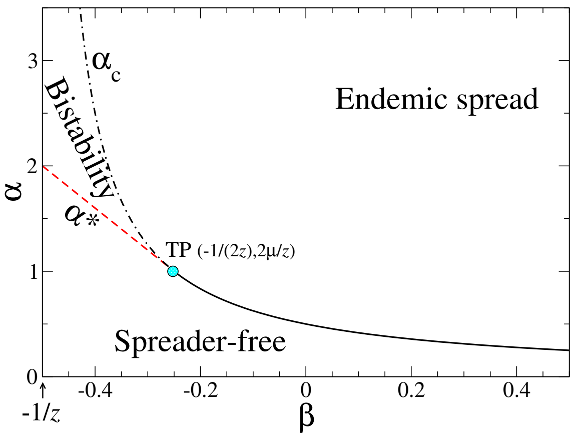

FIG. 1. Contagion diagram for the SIS model with linear synergy. The solid line represents the threshold $\alpha_c(\beta)$ for continuous transitions between the spreader-free and invasive endemic regimes. The circle labelled by TP indicates the tricritical point. The dot-dashed line corresponds to $\alpha_c(\beta)$ in the region with explosive transitions. The bi-stability region is bounded from below by the dashed line corresponding to the function $\alpha^*(\beta)$. Numerical values along the axes correspond to random z-regular graphs with $z = 2$ and $\mu = 1$. The horizontal axis shows only meaningful values of $\beta > -1/z$.

librium at y_- corresponds to the endemic state (the region above the dot-dashed line in Fig. 1). In the bi-stable regime with $\alpha \in (\alpha_*(\beta), \alpha_c(\beta))$, the mean-field system, depending on initial conditions, reaches the spreader-free regime, $y_{\rm sf}$, or the endemic regime, y_-. The dotted lines in Fig. 2(b) indicate the explosive transitions observed by increasing α from $\alpha < \alpha^*$ (up arrow) or decreasing from $\alpha > \alpha_{\rm c}$ (down arrow). A hysteresis loop of width $\alpha_{\rm c} - \alpha^*$ becomes wider as β becomes more negative.

MODELS WITH REMOVAL OF SPREADERS ON z-RANDOM REGULAR GRAPHS

In this section, we derive the general solution (Eq. (15) of the main text) for the mean-field models with removal of spreaders and illustrate its properties using the SIR model with linear synergistic transmission rate as a benchmark.

From Eqs. (11)-(13) of the main text and the definition of $\lambda_z(x) = \alpha\sigma_z(x)$, one obtains,

$$y = -\frac{1}{\alpha z \sigma_z(x) x}\frac{\mathrm{d}x}{\mathrm{d}t} = \frac{1}{\mu\gamma(x)}\frac{\mathrm{d}r}{\mathrm{d}t}\ . \tag{5}$$

Integrating the second equation in Eq. (5) over time in the interval $[0, t]$ leads to the following expression:

$$-\int_{x_0}^{x(t)} \frac{\gamma(x)}{\sigma_z(x) x}\mathrm{d}x = \frac{z\alpha}{\mu}\int_0^{r(t)} \mathrm{d}r\ . \tag{6}$$

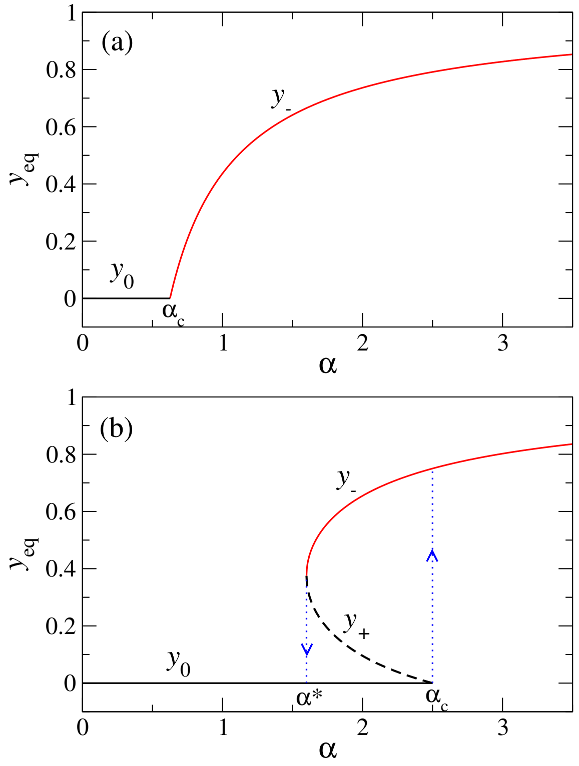

FIG. 2. Equilibrium concentration, $y_{\rm eq}$ ($y_{\rm eq} \in [0,1]$), of spreaders in SIS epidemics on random z-regular graphs with $z = 2$ and $\mu = 1$ *vs* inherent transmission rate, α. Linear synergy rates with (a) $\beta = -0.1 > \beta_{\rm tp}$ and (b) $\beta = -0.4 < \beta_{\rm tp}$, illustrate continuous and explosive transitions, respectively. In (b), the equilibrium concentration y_+ corresponds to unstable states (dashed line). The dotted vertical lines at $\alpha_{\rm c}$ and α^* indicate explosive transitions with increasing and decreasing α, respectively.

Here, we have assumed a population which initially consists of only ignorants and spreaders, *i.e.*, $r(0) = 0$, $x(0) = x_0 \le 1$ and $y(0) = 1 - x_0$. From Eq. (6), the concentration of removed individuals over time, $r(t)$, can be expressed as a function of the concentration of ignorants as follows:

$$r(t) = \frac{\mu}{\alpha z}\left[F_2(x_0) - F_2(x(t))\right]\ . \tag{7}$$

The function $F_2(x)$ is defined in Eq. (16) of the main text.

The fixed points of the system given by Eqs. (11)-(13) in the main text correspond to states without spreaders, $y = 0$. In general, any finite system with an initially positive concentration of spreaders, $y_0 > 0$, and positive removal rate, $\gamma(x) > 0$, evolves towards a fixed point with $y = 0$, $x = x_\infty$ and $r_\infty = 1 - x_\infty$. The condition $y = 0$ points out the end of the epidemic. Examples of the evolution of x and r are shown in Figs. 3 and 4 for the

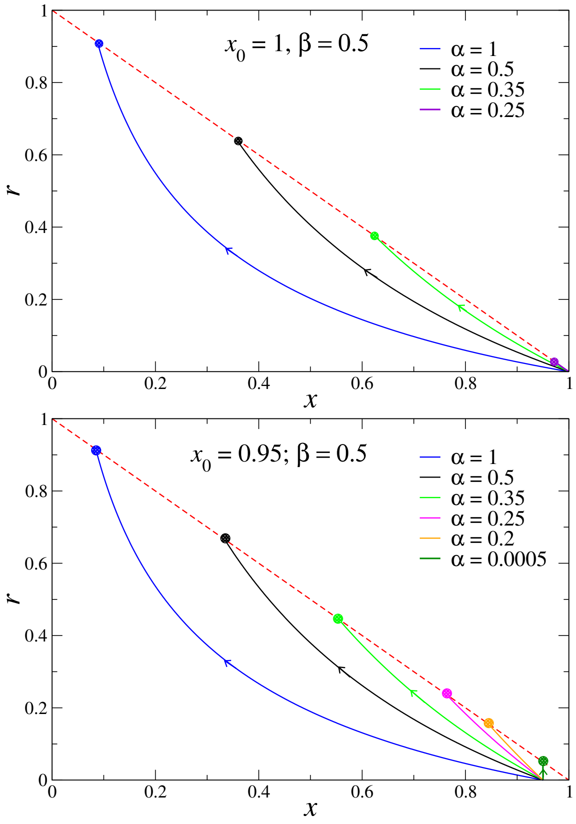

FIG. 3. Trajectories of the concentration of removeds and ignorants during SIR epidemics with $\mu = 1$ and linear synergy rate for $\beta = 0.5$ spreading on random z-regular graphs with $z = 2$. The initial concentration of ignorants is $x_0 = 1$ and $x_0 = 0.95$ in the upper and lower panel, respectively. Epidemics stop (circles) when the trajectories reach the dashed line corresponding to $r = 1 - x$. The final concentration of removeds, r_∞, increases smoothly with α in both panels.

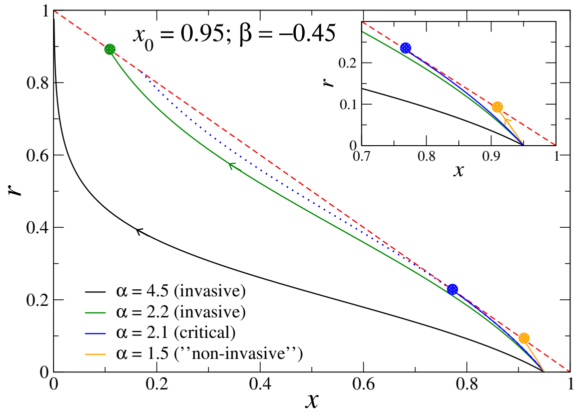

FIG. 4. Trajectories of the concentration of removeds and ignorants during SIR epidemics on random z-regular graphs with $z = 2$ for $x_0 = 0.95$, $\beta = -0.45$, $\mu = 1$ and linear synergy rate with $\beta = -0.45$. The dashed line shows the function $r = 1 - x$ giving the locus of concentrations at the end of epidemics. Circles indicate the final state of each trajectory at $r_\infty = 1 - x_\infty$. The final concentration of removeds, r_∞, changes abruptly with increasing α, thus indicating an explosive transition to large contagion. The explosion occurs at the critical value of $\alpha_c \simeq 2.1$. For this critical value, the blue dotted line shows solutions of Eq. (8) that are not reached during the epidemic because the epidemic (solid blue line) terminates at the point denoted by the blue circle which corresponds to the largest value of x_∞.

SIR model with linear synergy for several values of x_0, α and β. The value of the final concentration of ignorants, x_∞ (or removeds, $r_\infty = 1 - x_\infty$), depends in general on the initial concentration of ignorants, $x_0 = 1 - y_0$, the inherent transmission rate, α, as well as on the synergistic and recovery mechanisms encoded by the functions σ_z and γ, respectively. Such dependence can be recast from Eq. (7) in the implicit form given by Eq. (15) of the main text which we repeat here for convenience:

$$\alpha = f(x_\infty; x_0) \equiv \frac{\mu}{z(1 - x_\infty)} \left[F_2(x_0) - F_2(x_\infty)\right] \ . \qquad (8)$$

It is clear from Eq. (8) that systems characterised by a function $f(x_\infty; x_0)$ that decreases monotonically with x_∞ will exhibit continuous transitions from smaller to larger r_∞ (from larger to smaller x_∞) with increasing α. Examples of this type of behaviour of $f(x_\infty; x_0)$ are shown by the continuous lines in Fig. 5 for the SIR model with linear synergy rate. In contrast, discontinuous transitions can occur when $f(x_\infty; x_0)$ is not monotonic and it increases with x_∞ in some sub-interval of $(0, 1)$. In this case, Eq. (8) can have several solutions for x_∞ corresponding to several fixed points (cf. dashed lines in Fig. 5). The evolution given by Eqs. (11)-(13) in the main text is such that x decreases with time from x_0 and the system evolves towards the solution corresponding to the largest value of x_∞; the rest of solutions are not accessible to the system. The trajectories of the SIR model with linear synergy shown in Fig. 4 illustrate this behaviour. In particular, the trajectory for α_c shows both the reachable (continuous line) and unreachable (dotted line) solutions of Eq. (8).

As mentioned in the main text, the regimes with continuous and explosive transitions are separated by a critical regime for which $f(x_\infty; x_0)$ displays an inflection point at some value of $x_\infty = x_{\rm tp} \in (0, 1)$. This situation corresponds to the tricritical point discussed in the main text. At the inflection point,

$$\left.\frac{\partial f(x; x_0)}{\partial x}\right|_{x_{\rm tp}} = \left.\frac{\partial^2 f(x; x_0)}{\partial x^2}\right|_{x_{\rm tp}} = 0 \ . \qquad (9)$$

These conditions and definition of $f(x_\infty; x_0)$ given by Eq. (8) result in Eqs. (17) and (18) given in the main

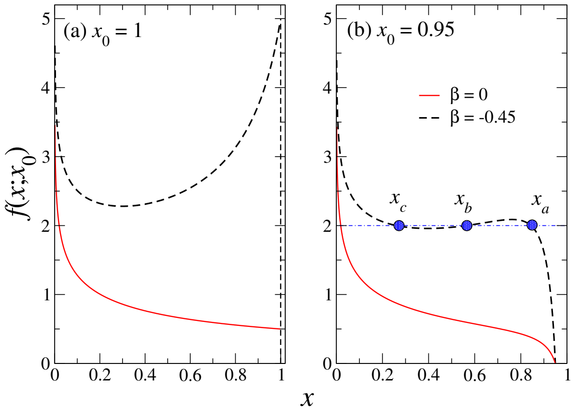

FIG. 5. Function $f(x; x_0)$ defined by Eq. (8) with x_∞ replaced by x, for (a) $x_0 = 1$ and (b) $x_0 = 0.95$ corresponding to SIR epidemics with linear synergy spreading on random regular graphs with $z = 2$. In both panels, the continuous and dashed lines correspond to $\beta = 0$ and $\beta = -0.45$, respectively. The horizontal dot-dashed line in (b) illustrates the solutions (circles) of Eq. (8) for $\alpha = 2$. The system evolves towards the largest solution and reaches the final concentration of ignorants $x_\infty = x_a$.

text for the tricritical point. For the SIR model with linear synergy, Eqs. (17)-(19) in the main text lead to the following relations at the tricritical point:

$$x_{\rm tp} = -\frac{1}{2z\beta_{\rm tp}} \;, \tag{10}$$

$$x_0 = -\frac{1}{z\beta_{\rm tp}(1 + e^{2(2z\beta_{\rm tp}+1)})} \;, \tag{11}$$

$$\alpha_{\rm tp} = -4\beta_{\rm tp}\mu \;. \tag{12}$$

Fig. 6 shows the phase diagram for the SIR model with linear synergistic transmission for two initial conditions: $x_0 = 1$ (i.e. a negligible initial concentration of infecteds, y_0) and $x_0 = 0.95$. For $x_0 = 1$, one obtains $\beta_{\rm tp} = -1/(2z)$ from Eq. (11) which leads to $x_{\rm tp} = 1$ and $\alpha_{\rm tp} = -2\mu/z$. The value of $\beta_{\rm tp}$ decreases with x_0 (see Fig. 7). This implies that social phenomena starting with a relatively large initial concentration of spreaders, $y_0 = 1 - x_0$, will require larger synergistic effects of the context in order for them to be explosive. However, explosive transitions exist for any initial conditions with $x_0 > 0$ since $\beta_{\rm tp}$ is finite for any $x_0 > 0$ (from Eq. (11), it is clear that $\beta_{\rm tp} \to -\infty$ only for $x_0 \to 0$.).

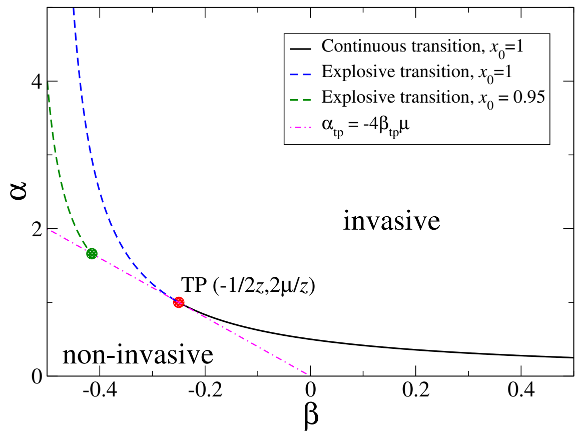

FIG. 6. Contagion phase diagram for the SIR model on random z-regular graphs. The continuous black line indicates the invasion threshold for continuous transitions observed for an initial concentration of ignorants, $x_0 = 1$. The solid straight line displays the locus of tricritical points given by Eq. (12). The blue and green dashed lines give the explosive invasion threshold for epidemics with $x_0 = 1$ and $x_0 = 0.95$, respectively. Numerical values along the axes correspond to random z-regular graphs with $z = 2$ and $\mu = 1$.

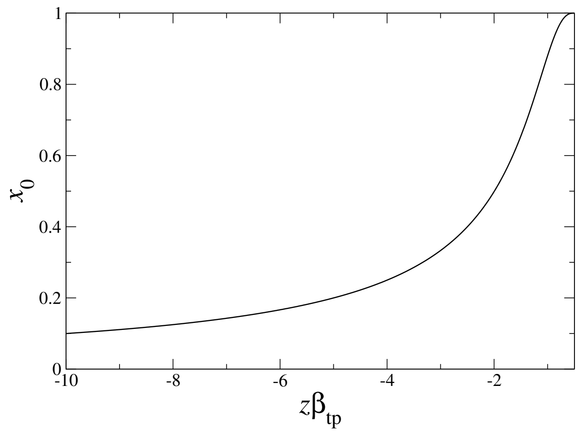

FIG. 7. Graphical representation of the dependence of $\beta_{\rm tp}$ on x_0 for the SIR model with linear synergy rate (cf. Eq. (11)).
